# Understanding Longitudinal Ecological Momentary Assessment Completion: Results From 12 Months of Burst Sampling in the TIME Study

**DOI:** 10.2196/67117

**Published:** 2025-10-22

**Authors:** Tyler Prochnow, Wei-Lin Wang, Shirlene Wang, Jixin Li, Alexander J Rothman, Stephen S Intille, Donald Hedeker, Genevieve F Dunton

**Affiliations:** 1 School of Public Health Texas A&M University College Station, TX United States; 2 Population and Public Health Sciences & Psychology University of Southern California Los Angeles, CA United States; 3 Khoury College of Computer Sciences and the Bouvé College of Health Sciences Northeastern University Boston, MA United States; 4 College of Liberal Arts University of Minnesota Minneapolis, MN United States; 5 Department of Public Health Sciences University of Chicago Chicago, IL United States

**Keywords:** ecological momentary assessment, longitudinal study, compliance, mobile health, young adults

## Abstract

**Background:**

Ecological momentary assessment (EMA) is a valuable method for capturing real-time data on behaviors and experiences in naturalistic settings. However, maintaining participant engagement in longitudinal (ie, multiburst) EMA studies remains challenging, particularly when collecting intensive data over extended periods. Understanding factors affecting completion rates is essential for designing more effective EMA protocols and interpreting results accurately.

**Objective:**

This study investigated factors influencing EMA completion rates in a 12-month intensive longitudinal study among young adults in the United States, examining both time-varying factors and stable individual characteristics.

**Methods:**

Young adults (N=246, ages 18-29 years) participated in the Temporal Influences on Movement and Exercise (TIME) study, responding to smartphone-based EMA prompts during biweekly measurement bursts (4-day periods of intensive sampling), with continuous passive data collection via smartwatches. Each burst included signal-contingent prompts delivered approximately once per hour during waking hours, resulting in an average of 12.1 (SD 1.3) prompts per day. Multilevel logistic regression models examined the effects of time-varying temporal factors (time of day, day of week, season, and time in study), contextual factors (phone screen status, phone usage, and location), behavioral factors (sleep duration, physical activity levels, and travel status), and psychological factors (momentary affect and stress) on prompt completion. Models also included time-invariant demographic characteristics (sex, race, ethnicity, education, and employment) and tested interactions between time in study and other predictors.

**Results:**

Mean completion rate was 77% (SD 13%). Hispanic participants showed lower odds of completion compared to non-Hispanic participants (odds ratio [OR] 0.79, 95% CI 0.63-0.99; *P*=.04) and employed participants were less likely to complete prompts than unemployed participants (OR 0.75, 95% CI 0.61-0.92; *P*<.01). Having the phone screen on at prompt delivery substantially increased completion odds (OR 3.39, 95% CI 2.81-4.09; *P*<.001), while being away from home reduced completion likelihood, with particularly low odds when at sports facilities (OR 0.58, 95% CI 0.47-0.74; *P*<.001) or restaurants and shops (OR 0.61, 95% CI 0.51-0.72; *P*<.001). Short sleep duration the previous night (OR 0.92, 95% CI 0.87-0.99; *P*=.02) and traveling status (OR 0.78, 95% CI 0.75-0.82; *P*<.001) were associated with lower completion odds. Higher momentary stress levels predicted lower completion of subsequent prompts (OR 0.85, 95% CI 0.78-0.93; *P*<.001). Completion odds declined over the 12-month study (OR 0.95, 95% CI 0.94-0.96; *P*<.001), with significant interactions between time in study and various predictors, indicating changing patterns of engagement over time.

**Conclusions:**

Findings highlight the dynamic nature of EMA engagement in longitudinal multiburst studies and underscore the importance of considering time-varying and time-invariant factors in study design and analysis. This study provides valuable insights for researchers designing intensive longitudinal studies in behavioral science and digital health. Potential strategies for optimizing EMA protocols could include tailoring prompt schedules to individual contexts and developing adaptive sampling techniques.

## Introduction

Traditional cross-sectional and retrospective self-report measures are prone to substantial limitations in studying health behaviors and psychological processes [1]. Cross-sectional designs only provide a snapshot, lacking insight into within-person variations over time and context [2,3]. Retrospective surveys require participants to summarize or recall experiences over lengthy periods, introducing recall biases that distort reports [2,3]. For example, physical activity levels likely vary within days and across days of the week in systematic ways obscured in generalized retrospective reports [3,4]. Aggregated retrospective summaries also fail to capture specific within-day or day-to-day fluctuations in behaviors, feelings, thoughts, and exposures that may be highly predictive of outcomes [3]. Traditional designs lacking temporal specificity severely limit understanding of the microtemporal relationships between psychological factors and behavioral choices hypothesized by many health behavior theories and models [1-3,5]. Intensive longitudinal designs using ecological momentary assessment (EMA) are needed to overcome these limitations and capture predictive within-day and day-to-day variations [1-3].

EMA overcomes many limitations of traditional designs by using repeated natural sampling of current behaviors, experiences, and contexts [1-3]. EMA prompts participants to provide real-time self-reports multiple times per day in natural environments, reducing recall biases [1-3]. Capturing microtemporal fluctuations via intensive longitudinal EMA enables examination of within-day and day-to-day variations in behaviors and putative causal factors, unavailable in traditional designs [1-3]. For example, EMA allows modeling of physical activity as a function of rapidly changing variables such as location, affect, and stress measured at the same momentary time scale [6-9]. EMA’s temporal precision permits study of directionality and time-varying effect magnitudes [10]. EMA’s ecological validity also facilitates idiographic analysis of dynamic relationships among variables unfolding uniquely for each person [10,11]. Thus, intensive longitudinal EMA provides new opportunities to understand microtemporal influences on health behaviors.

However, EMA is prone to missing data due to noncompletion with intensive sampling protocols [12,13]. This can bias results if missing data are systematic [12,13]. For example, individuals may be less likely to comply when under stress or engaging in unhealthy behaviors, distorting conclusions [12,13]. It is critical to maximize completion, which remains a challenge in long-term studies [14]. Completion rates vary widely in EMA research, ranging from 42% to 99% with a mean of 82% [12]. Lower completion is associated with minority ethnicity, low socioeconomic status, and mental health symptoms [12]. Additionally, time-varying factors such as affect, social context, and time of day have been found to predict EMA compliance [15]. Completion also decays over time, with gradual drop-offs or sudden declines [13]. This complicates separating true change from artifactual patterns. It is important to note that most EMA studies are relatively short-term, typically lasting from a few days to several weeks, with some extending to a few months [12]. Longer-term studies, particularly those extending beyond 6 months, are relatively rare in EMA literature. This limited duration of typical EMA studies restricts our understanding of long-term patterns in compliance and the sustainability of intensive longitudinal designs. Systematically missed EMA prompts reduce statistical power and can preclude multilevel modeling approaches common for EMA data [1,12,14,16]. Thus, optimizing and tracking completion is essential for valid EMA studies. Understanding factors influencing completion is critical for interpreting findings and guiding effective retention strategies. Further, most prior research on EMA completion has focused on single measurement burst or limited measurement burst designs [1,3,12]. Consequently, there is a significant gap in our understanding of EMA compliance in multimeasurement burst designs with frequent bursts, particularly regarding changes in completion rates over extended periods and interactions between time-varying and time-invariant factors across time [1,3,12]. This lack of knowledge presents a critical challenge for researchers aiming to implement long-term, intensive EMA protocols, as it limits our ability to anticipate and mitigate compliance issues in such designs.

The Temporal Influences on Movement and Exercise (TIME) study combines 12 months of smartwatch data collection and EMA in young adults [16]. Young adulthood is a high-risk period for declining physical activity and deteriorating sleep patterns [17,18]. The TIME study’s intensive EMA protocol also enables examining how psychological and contextual variables relate to completion. Specifically, the length of follow-up (across 12 months), in combination with passive data collection (accelerometer data collection), and frequent intensive EMA “bursts” represent advances in EMA research. For example, the study can evaluate whether EMA completion rates decay more steeply when participants report higher stress levels or more negative affect. Additionally, it can examine if factors such as sleep quality, physical activity levels, or social interactions interact with time to predict changes in compliance. Findings from these analyses may point to the need for adaptive EMA protocols that tailor prompt frequency, timing, or content based on detected psychological states, behaviors, or contextual factors, potentially improving long-term engagement and data quality. The 12-month duration also permits evaluating long-term patterns such as seasonal variations. Thus, the TIME study provides a unique opportunity to elucidate participation and engagement factors important for future intensive longitudinal research.

Therefore, this study aims to evaluate the TIME study’s EMA completion rates and predictors. Specifically, the purpose of this study was to examine whether time-varying temporal, contextual, behavioral, and psychological factors (eg, time of day, day of week, screen status, location, days enrolled in study, time elapsed from last active phone use, sleep duration the prior night, current day activity level, current day average stress, previous hour physical activity, previous hour affect state, and previous hour stress level) and time-invariant personal characteristics (eg, sex at birth, race, ethnicity, education, and employment status) were associated with EMA completion rates. Investigating these patterns through an unprecedented, year-long intensive protocol provides critical methodological insights into the feasibility, sustainability, and data quality of long-term EMA studies. These findings can guide interpretations of results from longitudinal multiburst EMA studies, as systematically missed EMA prompts may distort conclusions by underrepresenting critical moments or obscuring true temporal relationships between variables. Identifying factors linked to completion can inform refinements to future EMA protocols and retention strategies to enhance data quality and representativeness. By understanding predictors of nonresponse, researchers can develop targeted strategies to reduce systematic missingness, such as implementing adaptive sampling techniques or personalized engagement approaches. These refinements aim to preserve statistical power by maximizing response rates, particularly during critical periods or states prone to missingness, ultimately providing more valid and reliable results for understanding real-time health behaviors and experiences. This study represents an important step in elucidating the sustainability and validity of long-term intensive EMA for studying health behaviors, offering empirical evidence to support best practices for designing and implementing longitudinal multiburst EMA studies that balance data quality with participant burden and engagement.

## Methods

### Design

The TIME study used a 12-month intensive longitudinal multiburst design using personal smartphones to continuously monitor young adults’ health behaviors and associated psychological processes. As detailed in the study protocol [16], participants serve as their own controls in a within-subject case-crossover framework, capturing dynamic fluctuations in behaviors and predictors over time and context. Passive sensing via smartwatch and phone sensors provides continuous measurement of activity behaviors and contextual factors such as time and location hypothesized to reactively influence those behaviors [16]. Intermittently prompted EMAs on smartphones gather self-reports on reflective cognitive factors such as intentions and self-control. This innovative combination of continuous passive monitoring and bursting EMA surveys provides rich temporal data on factors influencing physical activity, sleep, and sedentary time. The extensive 12-month duration enabled studying maintenance as well as adoption of health behaviors.

### Ethical Considerations

The study was approved by the institutional review board at the University of Southern California (USC; HS-18-00605). The study was performed in accordance with the ethical standards as laid down in the Declaration of Helsinki and its later amendments. All participants provided informed consent to have their deidentified data published in journals. Participants were compensated up to US $100 for each 4-week period (US $1260 total). This included US $10 for each EMA burst period they completed, with at least 8 prompts per day. In addition, if a participant answered more than 11 EMA prompts on a given burst period day, the participant receives a US $5 bonus per day.

### Participants

The study recruited young adults across the United States (N=246). Inclusion and exclusion criteria were assessed by self-report during the screening process. Inclusion criteria for the study were as follows: (1) aged 18-29 years living in the United States, (2) intention to engage in recommended levels of moderate to vigorous physical activity (≥150 minutes/week moderate or ≥75 minutes/week vigorous intensity) within the next 12 months, (3) use an Android-based smartphone as their only primary personal mobile device with no intention to switch to a non-Android phone, (4) able to speak and read English, and (5) plan to reside in a home with Wi-Fi connectivity during the study period. Exclusion criteria were (1) physical or cognitive disabilities that prevent participation; (2) health issues that limit physical activity; (3) any diagnosed sleep disorders; (4) unable to wear a smartwatch or answer EMA surveys at home, work, school, or another location where a substantial amount of time is spent; (5) spends more than 3 hours/day on a typical weekday or weekend day driving; (6) owns an Android phone version 6.0 (or older), or if the app will not function on the phone due to other technical issues; (7) currently owns and wears a smartwatch; (8) uses a pay-as-you-go data plan or data plan with less than 2 gigabytes of data per month; or (9) currently pregnant. Participants were recruited regardless of baseline activity level. A variety of recruitment methods were used to recruit socioeconomically and racially or ethnically diverse young adults, including (1) sending emails to individuals enrolled in a USC longitudinal cohort study of young adults, (2) referrals from existing participants (word of mouth), and (3) contacting participants identified using ResearchMatch, a national health volunteer registry [19].

Of 246 participants fully enrolled in the TIME study, 3 were excluded due to missing key demographic information, specifically sex at birth and age, reducing the subject-level sample size to 243. Within this cohort, 29 participants were further removed from the analysis due to missing phone data, including screen state or phone usage, thereby decreasing the sample size to 214. Additionally, missing accelerometer data from the watch led to the exclusion of 5 participants, culminating in a sample of 209. Lastly, missing EMA data necessitated the removal of 2 participants, resulting in a final analytical sample of 207. See Multimedia Appendix 1 for a more detailed overview of participant exclusions.

### Protocol

Data were collected between March 2020 and August 2022. Due to health and safety concerns arising from the COVID-19 pandemic, all study procedures were conducted remotely. Potential participants filled out a web-based interest form to screen eligibility (n=1202). For eligible and interested potential participants, a videoconference orientation and consent session over Zoom (Zoom Communications) was then scheduled (n=645). This session involved reviewing all parts of the study, obtaining informed consent, and downloading the custom TIME study smartphone app onto the participant’s smartphone (n=332). During the orientation session, participants received instructions on how to use the study app to complete EMA surveys. During the following week, individuals participated in their first 4-day EMA measurement burst period (further described below), during which the TIME app triggered surveys once per hour during the participant’s waking hours. Participants who successfully completed at least 8 surveys per day during this first EMA measurement burst period were fully enrolled in the study and mailed a smartwatch (N=246). If completion was below 8 surveys/day for the first measurement burst period, participants were unenrolled from the study. Among the fully enrolled cohort (N=246), 228 participants remained active in the study during the first 3 months. By month 6, the number of participants had decreased to 186, and only 123 participants completed the full-year study. The overall attrition rate was 50%.

EMA data were collected using the custom TIME app developed for Android smartwatches and smartphones. While more information about the app can be found in the study protocol paper [16], the app consists of an auditory prompting with a smartphone notification, followed by a welcome screen, followed by EMA items with one item per screen, and lastly a thank you screen. The app was downloaded directly to a participant’s personal Android phone from the Google Play Store but was only accessible to authorized study participants. Once the participant received the smartwatch by mail, the TIME app was downloaded to the watch paired with the smartphone. EMA surveys were prompted in four ways: (1) daily sleep-wake time surveys to tailor prompt timing to participants' schedules, (2) end-of-day surveys each evening, (3) 4-day measurement burst periods every 2 weeks with signal-contingent prompting approximately every hour, and (4) context-sensitive surveys based on passive sensing of location and activity. Participants also completed web-based questionnaires at baseline, 6 months, and 12 months, and an exit interview. This study focused on the completion of the 4-day measurement burst EMA prompts.

EMA measurement bursts were scheduled every 2 weeks, resulting in up to 26 burst periods throughout the year-long study, totaling 104 days of intensive data collection per participant. The burst periods were randomly scheduled but structured to always include 2 weekdays and 2 weekend days, with a minimum of 7 days between each burst. Each burst encompassed 4 consecutive days of data collection, with signal-contingent prompts occurring multiple times daily. These prompts were randomly triggered approximately once per hour during participants' waking hours, specifically between 10 and 50 minutes past each hour to prevent consecutive prompts from occurring too close together. Participants received a notification via the TIME app 1 day before each burst period was set to begin. This notification provided participants with a one-time option to delay the start of the burst by 2 days if needed.

To encourage ongoing participation and attentiveness, the researchers implemented several engagement strategies, including (1) texting participants when their EMA completion declined, (2) providing financial incentives based EMA completion, (3) allowing participants with high completion (ie, wearing the smartwatch for more than 23 hours/day on 24 days/month, answering more than 24 of the end-of-day EMA smartphone prompts/month, answering more than 11 EMA smartphone prompts/day during measurement bursts, and answering more than 50% of the micro-EMA questions prompted on the smartwatch) to keep their smartwatch at the end of the study, (4) sending quarterly participant newsletters, and (5) providing a report-back of individual data to participants who completed the study. Participants were oriented to the study protocol and given a number and instructed to text the study staff with any questions, concerns, or technical issues. On a weekly basis, staff review data uploaded to the study server and contact participants by email or SMS text messages, which is another avenue to address technical issues. After completing each EMA prompt, participants were shown a humorous and lighthearted “thank you” message. A bank of 250 unique messages was used to maintain novelty throughout the study. On a weekly basis, staff review data uploaded to the study server and contact participants by email or SMS text message in the case of missing data to encourage compliance and address technical issues. Additionally, 20% of the EMA prompts included attention check questions. These questions were designed to be both entertaining and unambiguous, allowing researchers to assess whether participants were paying attention and responding thoughtfully to the EMA questions. More information regarding the attention check questions as well as a qualitative evaluation of participant engagement can be found elsewhere [20].

### Activity Monitoring

Raw accelerometer data (using the embedded triaxial accelerometer) were collected on the phone and watch at ~50 Hz continuously. After data collection, the raw accelerometer data from the smartwatch were processed using the monitor-independent movement summary (MIMS) unit algorithm [21]. The MIMS-unit algorithm computes a summary of total movement within an epoch and has been used to identify percentile cutoffs for physical activity among young adults at a population scale [22]. MIMS-unit values were calculated for 1-second epochs for all the raw acceleration data collected from the wrists.

### Location Sensing

Location data (latitude and longitude) were gathered once per minute using the built-in GPS features on the phone. By the end of each day, the location data were converted to location clusters, which were then used for self-reported location labeling. First, at midnight, the app clustered the entire day’s location data using the DBSCAN (density-based spatial clustering of applications with noise) algorithm [23]. Then, for each day, the clustered points were combined with the previous day’s cluster points and were clustered again using DBSCAN to generate location clusters for the participant, but this time considering all clusters identified since the start date. A cluster was formed when there were at least 5 stay points at that location; a stay point was defined as spending at least 5 minutes continuously in one 10 m radius (4th decimal precision for longitude/latitude). The global clusters were saved as polygons of bounding points (using the concave hull [24]).

In real time, the app also checked if the current location was within an already identified cluster for at least 3 minutes; if this condition was satisfied, the phone presented a location question to gather semantic labels. If participants answered this survey, then the label was saved for the cluster. The question was presented again for this cluster until the labeled cluster was *confirmed* (when 80%+ of the labels for that location were the same, and at least 4 labels were reported). Once the label was confirmed for a cluster, no location question was prompted for this cluster again until an additional 60 days had passed, after which the question was presented again. If the location was again confirmed, another 60 days would pass without questions about the cluster; otherwise, more questions were asked until a label was again confirmed.

### Measures

Table 1 provides an overview of the measures used for analysis in this study, divided by the type of variable.

**Table 1:** Variables used in the analysis of ecological momentary assessment prompt completion.

Type	Variable	Collection method
Time-varying temporal	Time of the day Type of day (weekday/weekend) Months enrolled in the study Season (Reference=Summer)	EMA^a^
Time-varying contextual	Active phone screen state (Reference=Phone not actively being used at time of prompt) Phone usage (time since last phone use) Physical location	EMA
Time-varying behavioral	Sleep duration the prior night Previous 10-minute physical activity (as an ordinal and continuous variable) Daily status (eg, sick or traveling)	Accelerometer EMA
Time-varying psychological	Affect state on previous prompt Stress level on previous prompt	EMA
Time-invariant demographics	Sex at birth Age Race Ethnicity Education Employment status	Questionnaire

^a^EMA: ecological momentary assessment.

#### EMA Completion

The primary outcome of interest is prompt completion (ie, 1=completed and 0=not completed) as defined by responding and completing the full survey within the 10 minutes of the initial prompt. If a response is not provided, up to 2 reprompts will be provided at 5-minute intervals. After 10 minutes, the EMA survey becomes inaccessible and is recorded as not completed. The completion rate was measured at the prompt level and represents the availability of data.

#### Time-Varying Factors

##### Temporal

Temporal covariates were measured to examine associations with prompt completion.

- Time of the Day:
  Time of the day was grouped into 4 categories: “morning” (8 AM to 12 noon), “afternoon” (12 noon to 4 PM), “evening” (4 PM to 8 PM), and “night” (8 PM to midnight). Time of the day was then dummy coded with “morning” as the reference category.
- Type of Day:
  Each day was coded as a weekday or a weekend day (Saturday and Sunday) as a categorical variable. The type of day was then dummy coded with weekday as reference.
- Months Enrolled in the Study:
  In this study, the number of months in the study was used as a predictor for nonresponse.
- Season:
  Season of the year (summer, spring, fall, and winter) was used to better understand the role of seasonality on prompt response, with summer serving as the reference category.

##### Contextual

Time-varying contextual covariates were measured to examine associations with prompt completion.

- Active Phone Screen State:
  For each survey, a variable was created to display whether a prompt occurred when the phone was in active use with the screen on. Phone screen status was dummy coded with screen off as reference (screen off=0 and screen on=1).
- Time Since Last Phone Use:
  The time difference between a given prompt and the last active phone screen locking event (ie, when the active use session ends) was calculated as time since last phone use. However, a ceiling of 60 minutes was set, based on the prior work, to avoid long time gaps due to sleep time or the device being off. If the prompt occurred during active phone use, then the time difference was logged as 0 minutes.
- Location:
  Participants provided semantic location labels. The question asked, “Where are you right now?” with 21 answer options: “home,” “work,” “school/college,” “park/playground,” “sports field/court/golf course,” “gym/health club/fitness center,” “friend’s/romantic partner’s place,” “family member’s place,” “restaurant/bar/café,” “store/shopping venue,” “church/place of worship,” “movie theatre/entertainment venue,” “beach/pool,” “transit center/bus stop,” “medical clinic/hospital,” “in car/vehicle/train,” “salon/barber/spa,” “library/museum,” “gas station/convenience store,” “parking lot/structure,” “hotel/motel,” and “other.” First, all location points within a 5-minute interval were gathered, and then the point closest in time to the prompt was selected. Finally, among all semantic location labels reported for this cluster, the one with the largest count was picked. Only the most frequently occurring labels were included: “home of participant,” “home of relatives or friends,” “work or school,” “shop or restaurant,” and “indoor or outdoor sport place.” Finally, all other location labels that did not occur frequently were tagged as “other” locations. The location dummy variable was coded with “home” as a reference category.

##### Behavioral

Time-varying behavioral covariates were also explored.

- Sleep Duration the Prior Night:
  Sleep duration was calculated as the time difference between the retrospective wake-up time on the day of the survey and the retrospective bedtime on the preceding night.
- Daily Status:
  Daily status was meant to code whether the participant noted that they were sick or traveling on that day as these states can affect responsiveness to EMA prompts.
- Previous 10 Minutes Physical Activity:
  MIMS in the previous 10 minutes were calculated to determine proximal activity predictors. These data were analyzed in 2 different forms to examine the relationship between recent physical activity and EMA completion. First, we treated MIMS as an ordinal categorical variable to test for potential threshold effects and nonlinear relationships between activity intensity and EMA completion. We established activity intensity categories based on the distribution of MIMS values in our sample and aligned with conceptually meaningful activity intensity levels: “missing data”: no accelerometer data available; “less than 10 MIMS per minute”: representing primarily sedentary behavior and light physical activity; “10-15 MIMS per minute”: representing moderate-intensity physical activity; “greater than 15 MIMS per minute”: representing vigorous-intensity physical activity. These cut-points were determined through exploratory analysis of our dataset, examining the distribution of MIMS values during known activity types reported by participants, and selected to create meaningful distinctions between different activity intensities relevant to our research questions [21,25]. Second, we also analyzed MIMS as a continuous variable to examine the potential linear relationship between activity intensity and EMA completion probability. This dual analytical approach allowed us to determine whether physical activity's effect on EMA completion follows a threshold pattern (better captured by distinct categories) or a dose-response relationship (better captured by a continuous measure). Additionally, using both approaches serves as a sensitivity analysis to ensure our findings are robust across different analytical methods.

##### Psychological

Time-varying psychological factors were also explored.

- Previous Prompt Positive Affect State:
  Positive affect was measured using a set of questions asking participants to rate their current mood, such as happy, energetic, and relaxed, on a scale from 1 (not at all) to 5 (extremely), and we created a composite score out of these 3 items for the positive affect at each EMA prompt.
- Previous Prompt Stress Level:
  Stress was assessed using a single-item measure that asked participants to rate their current stress level on a scale from 1 (not at all stressed) to 5 (extremely stressed) at each EMA prompt.

#### Time-Invariant Covariates

Time-invariant covariates were recorded using a self-reported questionnaire at the beginning of the study. Sex at birth (binary variable: Female=1 Ref=Male), race (categorical variable), education (ordinal variable), and employment status (binary variable: 1=employed, 0=other) were all used in this study to examine completion as they have been previously used in previous studies.

### Data Analysis

Data analysis was conducted using multilevel logistic regression models to examine predictors of EMA prompt completion (yes/no) over the 12-month study period. This approach was chosen to account for the nested structure of the data, with multiple observations (level 1) clustered within participants (level 2). Five separate models were constructed to investigate different sets of predictors. Separate models were used due to the large number of predictors and the potential for multicollinearity among certain variables. This approach allowed us to examine specific sets of predictors and interactions in detail while maintaining model stability and interpretability. Additionally, using separate models enabled us to explore different aspects of EMA completion behavior (eg, contextual factors and psychological states) without overfitting the data or compromising statistical power. The use of multiple models also provided means to cross-validate findings and assess the robustness of predictor effects across different model specifications. Model 1 focused on demographics (sex, age, race, ethnicity, education, and employment status), time in study (both between- and within-subject effects), and date and time variables (season, day of week, and time of day). Model 2a examined demographics, time in study, phone status (screen on/off and usage in prior hour), physical context (location), and physical activity (categorized into levels based on MIMS units). Model 2b was similar to 2a but treated physical activity as a continuous variable. Model 3a investigated demographics, time in study, sleep duration (categorized as short, normal, or long), daily status (sick or traveling), and lagged positive affect. Model 3b was similar to 3a but replaced positive affect with stress. All models included both between-subject and within-subject effects for time-varying predictors, representing their deviations from the grand and subject mean, respectively. This allows for the separation of between-person differences and within-person fluctuations in the predictors. Interaction terms between time spent in the study and other predictors (eg, sex, phone status, affect, and stress) were also included in each model to examine how the influence of various factors on EMA completion might change over the course of the study. We examined interactions between time spent in the study (both between-subject and within-subject effects) and a range of factors. These included demographic characteristics such as participant sex, contextual factors like phone screen status before reci and phone usage in the prior hour, and behavioral measures such as physical activity levels. Additionally, we investigated how time spent in the study might moderate the effects of psychological factors on EMA completion, focusing on interactions with positive affect and stress levels. All of these interaction terms were included to assess whether the influence of these factors on EMA completion rates changed as participants progressed through the year-long study period. By incorporating these interactions, we aimed to capture the dynamic nature of factors influencing EMA compliance over time and to identify any temporal patterns in participant engagement with the study protocol. The outcome variable in all models was the binary completion status of each EMA prompt (1=completed and 0=not completed). Odds ratios (ORs) with 95% CIs were calculated for each predictor, representing the change in odds of EMA completion associated with a one-unit increase in the predictor.

## Results

### Descriptive Results

Among the final analytical sample (n=207), 55.56% (n=115) identified as female, 43.48% (n=90) as White, and 30.43% (n=63) identified as Hispanic. In terms of education and employment, 45.41% (n=94) had a college degree or higher, and 59.9% (n=124) were fully employed. The mean age of the participants was 23.45 (SD 3.12) years.

### Prompt Completion Descriptive Statistics

This intensive design resulted in an average of 12.1 (SD 1.3) prompts per participant per day, and 939.7 (SD 444.7) prompts per participant over the course of the whole survey (up to 1 year). On average, each participant had 51 (SD 30.5) days nested within 15.5 (SD 8.4) bursts. The overall mean completion rate for participants in the analytical sample was 0.77 (SD 0.13), indicating 77% of prompts were completed.

### Factors Predicting Prompt EMA Completion

Multilevel logistic regression analysis was conducted to examine time-varying and time-invariant predictors of EMA prompt completion over the 12-month study period. All models can be found in Table 2.

**Table 2:** Multilevel logistic regression models predicting EMA completion rates in a 12-month longitudinal study.

		Model 1			Model 2a			Model 2b			Model 3a			Model 3b	
Predictor		OR^a^ (95% CI)	*P* value	OR (95% CI)		*P* value	OR (95% CI)		*P* value	OR (95% CI)		*P* value	OR (95% CI)		*P* value
Intercept		3.80 (2.51-5.75)	<.001	3.40 (1.66-6.98)		<.01	4.57 (1.54-13.54)		<.01	3.78 (1.20-11.91)		.02	5.84 (2.95-11.56)		<.001
**Time-invariant demographic**															
	Sex (Female=1; reference: male)	0.78 (0.48-1.26)	.30	0.78 (0.63-0.96)		.02	0.73 (0.58-0.92)		<.01	0.84 (0.69-1.02)		.08	0.86 (0.72-1.03)		.13
	Age (Centering to 25 years)	0.97 (0.93-1.01)	.17	0.97 (0.93-1.01)		.14	0.97 (0.93-1.02)		.26	0.98 (0.94-1.02)		.26	0.98 (0.94-1.02)		.26
	Race: African American (reference: White)	1.10 (0.78-1.55)	.59	0.88 (0.60-1.29)		.50	0.85 (0.57-1.28)		.43	1.09 (0.77-1.55)		.61	1.06 (0.75-1.50)		.73
	Race: Asian (reference: White)	0.74 (0.58-0.95)	.02	0.74 (0.56-0.98)		.03	0.73 (0.54-0.99)		.04	0.72 (0.56-0.92)		<.01	0.73 (0.57-0.93)		.01
	Race: Other (reference: White)	0.88 (0.68-1.14)	.34	0.78 (0.59-1.04)		.09	0.72 (0.53-0.97)		.03	0.86 (0.67-1.11)		.26	0.87 (0.68-1.12)		.29
	Hispanic ethnicity (reference: Non-Hispanic)	0.79 (0.63-0.99)	.04	0.78 (0.61-1.00)		.05	0.76 (0.58-0.99)		.04	0.79 (0.64-0.99)		.04	0.77 (0.63-0.94)		.02
	Education: College and above (reference: less than college level)	1.22 (0.97-1.54)	.09	1.39 (1.08-1.79)		.01	1.35 (1.03-1.77)		.03	1.22 (0.96-1.53)		<.01	1.20 (0.96-1.52)		.11
	Employ status: Employed (reference: other)	0.75 (0.61-0.92)	<.01	0.72 (0.57-0.91)		<.01	0.67 (0.52-0.86)		<.01	0.77 (0.63-0.95)		.02	0.77 (0.63-0.94)		.01
**Time-varying temporal**															
	Month-stayed in study (BS^b^)	1.07 (0.99-1.15)	.07	1.16 (1.01-1.33)		.03	1.12 (0.92-1.36)		.26	1.11 (0.89-1.38)		.36	1.09 (0.96-1.23)		.21
	Month-stayed in study (WS^c^)	0.95 (0.94-0.96)	<.001	0.99 (0.97-1.01)		.22	0.97 (0.95-0.99)		.01	0.93 (0.91-0.95)		<.001	0.95 (0.93-0.97)		<.001
	Season: spring (reference: summer)	1.05 (1.00-1.10)	.04	—^d^		—	—		—	—		—	—		—
	Season: fall (reference: summer)	1.01 (0.97-1.06)	.60	—		—	—		—	—		—	—		—
	Season: winter (reference: summer)	1.06 (1.01-1.11)	.01	—		—	—		—	—		—	—		—
	Day of week: weekend (reference: weekday)	1.00 (0.97-1.03)	.98	—		—	—		—	—		—	—		—
	Time of day: afternoon (reference: morning)	1.01 (0.97-1.06)	.63	—		—	—		—	—		—	—		—
	Time of day: evening (reference: morning)	0.98 (0.94-1.03)	.54	—		—	—		—	—		—	—		—
	Time of day: night (reference: morning)	0.76 (0.62-0.93)	<.01	—		—	—		—	—		—	—		—
**Time-varying contextual**															
	Screen on before answering the survey (reference: screen off)	—	—	3.39 (2.81-4.09)		<.001	3.85 (3.64-4.08)		<.001	—		—	—		—
	Phone usage in prior hour (BS)	—	—	1.02 (0.72-1.45)		.89	0.76 (0.64-0.90)		<.01	—		—	—		—
	Phone usage in prior hour (WS)	—	—	0.80 (0.77-0.83)		<.001	0.82 (0.81-0.83)		<.001	—		—	—		—
	Missing location (reference: home)	—	—	0.53 (0.51-0.56)		<.001	0.54 (0.51-0.57)		<.001	—		—	—		—
	Home of relatives or friends (reference: home)	—	—	0.84 (0.77-0.92)		<.001	0.83 (0.74-0.92)		<.001	—		—	—		—
	Work or school (reference: home)	—	—	0.70 (0.65-0.75)		<.001	0.69 (0.63-0.75)		<.001	—		—	—		—
	Shop or restaurant (reference: home)	—	—	0.61 (0.51-0.72)		<.001	0.62 (0.51-0.75)		<.001	—		—	—		—
	Indoor or outdoor sport place (reference: home)	—	—	0.58 (0.47-0.72)		<.001	0.58 (0.46-0.75)		<.001	—		—	—		—
	Others (reference: home)	—	—	0.60 (0.48-0.74)		<.001	0.53 (0.41-0.67)		<.001	—		—	—		—
**Time-varying behavioral**															
	MIMS^e^ in prior 10 minutes: Missing	—	—	1.30 (1.23-1.38)		<.001	—		—	—		—	—		—
	MIMS in prior 10 minutes: Less than 10 MIMS per minute	—	—	1.35 (1.28-1.43)		<.001	—		—	—		—	—		—
	MIMS in prior 10 minutes: Equal or more than 10 and Less than 15 MIMS per minute	—	—	1.30 (1.22-1.39)		<.001	—		—	—		—	—		—
	MIMS in prior 10 minutes: Equal or more than 15 and Less than 20 MIMS per minute	—	—	1.16 (1.08-1.25)		<.001	—		—	—		—	—		—
	MIMS in prior 10 minutes (BS)	—	—	—		—	1.29 (0.53-3.18)		.57	—		—	—		—
	MIMS in prior 10 minutes (WS)	—	—	—		—	0.91 (0.82-1.01)		.06	—		—	—		—
	Sleep short (Less than 6 hours; reference: sleep between 6 and 11 hours)	—	—	—		—	—		—	0.92 (0.87-0.99)		.02	0.92 (0.86-0.98)		.02
	Sleep long (More than 11 hours; reference: sleep between 6 and 11 hours)	—	—	—		—	—		—	1.01 (0.95-1.08)		.74	1.01 (0.95-1.08)		.72
	No sleep data (reference: sleep between 6 and 11 hours	—	—	—		—	—		—	0.96 (0.91-1.01)		.08	0.96 (0.91-1.01)		.08
	Sick (reference: nonsick)	—	—	—		—	—		—	1.04 (0.96-1.12)		.36	1.04 (0.96-1.13)		.36
	Traveling (reference: nontraveling)	—	—	—		—	—		—	0.78 (0.75-0.82)		<.001	0.78 (0.75-0.81)		<.001
	Time-varying psychological														
	Positive affect (BS)	—	—	—		—	—		—	1.07 (0.73-1.58)		.73	—		—
	Positive affect (WS)	—	—	—		—	—		—	0.97 (0.87-1.09)		.61	—		—
	Stressed (BS)	—	—	—		—	—		—	—		—	0.89 (0.66-1.22)		.48
	Stressed (WS)	—	—	—		—	—		—	—		—	0.85 (0.78-0.93)		<.001
**Interactions**															
	Month-stayed in study (BS) × sex	1.02 (0.92-1.13)	.72	—		—	—		—	—		—	—		—
	Month-stayed in study (WS) × sex	0.99 (0.98-1.00)	<.01	—		—	—		—	—		—	—		—
	Month-stayed in study (BS) × screen on	—	—	1.02 (0.99-1.06)		.23	—		—	—		—	—		—
	Month-stayed in study (WS) × screen on	—	—	0.97 (0.95-0.99)		<.001	—		—	—		—	—		—
	Month-stayed in study (BS) × phone usage in prior hour (BS)	—	—	0.94 (0.87-1.01)		.08	—		—	—		—	—		—
	Month-stayed in study (WS) × phone usage in prior hour (BS)	—	—	0.98 (0.97-0.99)		<.001	—		—	—		—	—		—
	Month-stayed in study (BS) × phone usage in prior hour (WS)	—	—	1.00 (0.99-1.01)		.37	—		—	—		—	—		—
	Month-stayed in study (WS) × phone usage in prior hour (WS)	—	—	1.00 (1.00-1.01)		<.01	—		—	—		—	—		—
	Month-stayed in study (BS) × MIMS in prior 10 minutes (BS)	—	—	—		—	0.98 (0.82-1.17)		.86	—		—	—		—
	Month-stayed in study (WS) × MIMS in prior 10 minutes (BS)	—	—	—		—	0.99 (0.97-1.01)		.25	—		—	—		—
	Month-stayed in study (BS) × MIMS in prior 10 minutes (WS)	—	—	—		—	1.00 (0.98-1.02)		.83	—		—	—		—
	Month-stayed in study (WS) × MIMS in prior 10 minutes (WS)	—	—	—		—	1.00 (0.99-1.01)		.73	—		—	—		—
	Month-stayed in study (BS) × positive affect (BS)	—	—	—		—	—		—	0.99 (0.92-1.07)		.75	—		—
	Month-stayed in study (WS) × positive affect (BS)	—	—	—		—	—		—	1.01 (0.99-1.01)		.18	—		—
	Month-stayed in study (BS) × positive affect (WS)	—	—	—		—	—		—	1.01 (0.99-1.03)		.23	—		—
	Month-stayed in study (WS) × positive affect (WS)	—	—	—		—	—		—	1.00 (0.99-1.01)		.82	—		—
	Month-stayed in study (BS) × stressed (BS)	—	—	—		—	—		—	—		—	0.99 (0.93-1.05)		.76
	Month-stayed in study (WS) × stressed (BS)	—	—	—		—	—		—	—		—	1.00 (0.99-1.01)		.45
	Month-stayed in study (BS) × stressed (WS)	—	—	—		—	—		—	—		—	1.02 (1.01-1.04)		<.01
	Month-stayed in study (WS) × stressed (WS)	—	—	—		—	—		—	—		—	1.00 (1.00-1.01)		.76

^a^OR: odds ratio.

^b^BS: between subject.

^c^WS: within subject.

^d^Not applicable.

^e^MIMS: monitor-independent movement summary.

#### Time-Invariant Demographic Factors

Several time-invariant demographic factors were found to significantly predict EMA completion odds across multiple models. In Model 2a, sex at birth played a role, with females showing lower odds of completion compared to males (OR 0.78, 95% CI 0.63-0.96; *P*=.02). While no significant effect of age was observed in any model, race and ethnicity were important predictors. Model 1 showed that Asian participants had lower odds of completion compared to White participants (OR 0.74, 95% CI 0.58-0.95; *P*=.02), and Hispanic participants showed lower odds of completion compared to non-Hispanic participants (OR 0.79, 95% CI 0.63-0.99; *P*=.04). Education level emerged as a positive predictor in Model 2a, with higher education levels (college and above) associated with increased odds of EMA completion (OR 1.39, 95% CI 1.03-1.77; *P*=.01). In contrast, employment status was negatively associated with completion across models, as fully employed individuals showed lower odds of completion than those who were not (OR 0.75, 95% CI 0.61-0.92, *P*>.01 in Model 1).

#### Time-Varying Temporal Factors

Multiple regression analyses revealed several significant time-varying temporal factors influencing EMA completion odds. In Model 1, time of day played a crucial role, with nighttime associated with lower odds of completion compared to morning hours (OR 0.76, 95% CI 0.62-0.93; *P*>.01). Interestingly, no significant difference was found between weekdays and weekends across all models, suggesting that the day of the week did not statistically impact completion rates. Seasonal effects were observed in Model 1, with higher odds of completion in spring (OR 1.05, 95% CI 1.00-1.10; *P*=.04) and winter (OR 1.06, 95% CI 1.01-1.11; *P*=.01) compared to summer. The duration of participation in the study showed a significant within-subject decrease in completion over time (OR 0.95, 95% CI 0.94-0.96, *P*<.001 in Model 1), indicating declining completion odds as participants progressed through the study.

#### Time-Varying Contextual Factors

Models 2a and 2b highlighted the importance of phone-related factors for EMA completion. Having the phone screen on before answering the survey significantly increased the odds of completion (OR 3.39, 95% CI 2.81-4.09, *P*<.001 in both models), while higher within-subject phone usage in the prior hour decreased completion odds (OR 0.80, 95% CI 0.77-0.83, *P*<.001 in Model 2a; OR 0.82, 95% CI 0.81-0.83, *P*<.001 in Model 2b). Physical location was also a significant predictor in these models, with being outside the home generally associated with lower completion odds. For example, compared to being at home, participants were less likely to complete EMAs when at work or school (OR 0.70, 95% CI 0.65-0.75, *P*<.001 in Model 2a).

#### Time-Varying Behavioral Factors

The study found significant associations between behavioral factors and EMA completion odds across different models. In Models 3a and 3b, sleep duration from the prior night emerged as an important predictor, with short sleep duration (less than 6 hours) associated with lower completion odds (OR 0.92, 95% CI 0.87-0.99; *P*=.02) compared to normal sleep duration. Model 2a examined physical activity levels in the 10 minutes preceding the EMA prompt, finding that lower activity levels were generally associated with higher completion odds compared to high activity levels (ORs ranging from 1.16 to 1.35, all *P*<.001). However, when physical activity was used as a continuous variable (Model 2b), no significant effects were observed. Models 3a and 3b found that traveling was associated with decreased completion odds (OR 0.78, 95% CI 0.75-0.82, *P*<.001 in both models) although no significant effect was found for being sick.

#### Time-Varying Psychological Factors

Affective states and stress levels from the previous prompt showed significant associations with completion odds in Models 3a and 3b. Model 3a indicated that neither between-subject nor within-subject positive affect was statistically associated with completion odds, suggesting that the experience of positive emotions at the prior EMA prompt may not influence subsequent EMA completion behavior. However, Model 3b showed that within-subject stress levels had a significant negative association with odds of completing the subsequent EMA prompt (OR 0.85, 95% CI 0.78-0.93; *P*<.001), and it implied that participants were less likely to complete EMA surveys when experiencing elevated stress during the prior EMA prompt.

#### Interactions

**Figure 1:**
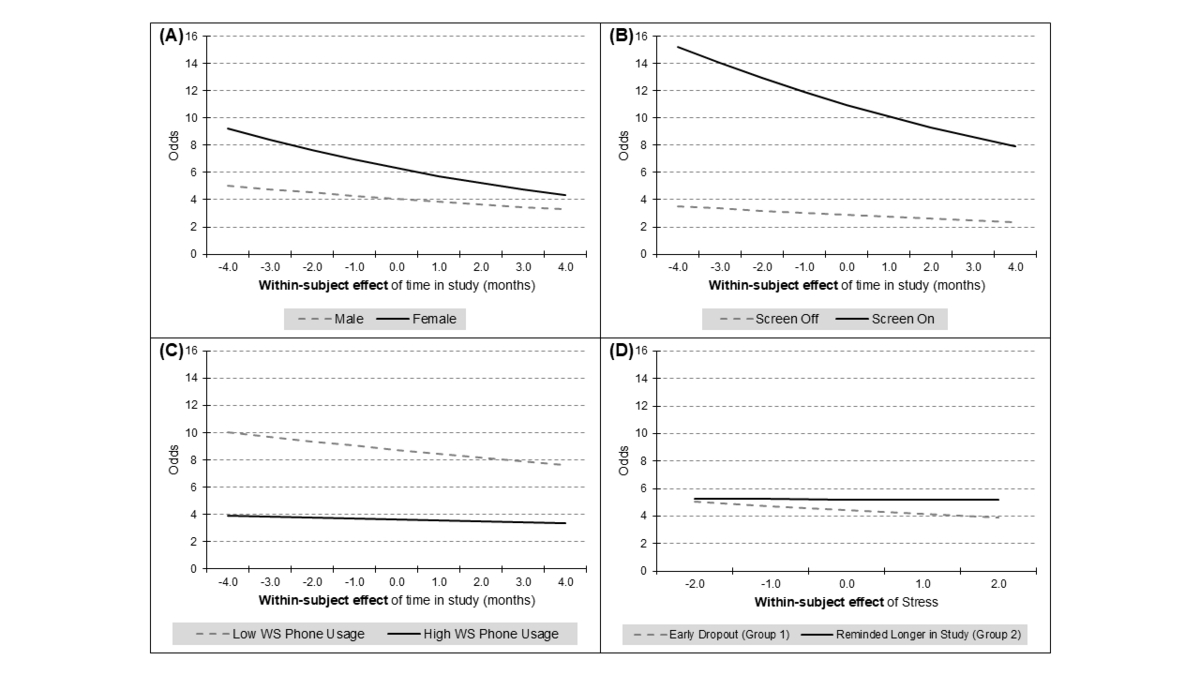
Interaction effects of key predictors and study duration on EMA completion. These plots illustrate the interaction effects between key predictors and study duration on momentary EMA completion odds. (A) Interaction effect between sex and within-subject time duration in study (ie, the deviation from each participant’s average month-stayed in the study) on completion odds as analyzed in Model 1. (B) Interaction effect between phone screen state and within-subject time duration in study on completion odds as examined in Model 2a. (C) Interaction effect between within-subject phone usage (ie, the deviation score from each participant’s average phone usage) and within-subject time duration in study on completion odds, also in Model 2a. (D) Interaction effect between within-subject momentary stress (ie, the deviation score from each participant’s average stress) and between-subject study duration (ie, early dropout vs reminded longer in study) on completion odds as detailed in Model 3b. EMA: ecological momentary assessment; WS: within subject.

The analysis revealed several significant interaction effects across different models, providing insights into how the influence of various factors on EMA completion odds changed over the course of the study. In Model 1, an interaction effect was observed between participant sex and within-subject time spent in the study, suggesting that females may experience a slightly faster decline in survey completion odds as the study progresses, relative to males (OR 0.99, 95% CI 0.98-1.00; *P*>.01; see Figure 1A). Model 2a showed interaction effects between screen status and within-subject time spent in the study (OR 0.97, 95% CI 0.95-0.99; *P*<.001), indicating that while having the phone screen on substantially increased completion odds initially, this positive effect diminished over time in the study (see Figure 1B). Similarly, the interaction between phone usage in the prior hour and within-subject time in the study (OR 0.98, 95% CI 0.97-0.99; *P*<.001) suggests that the negative impact of recent phone usage on completion odds became less pronounced as participants progressed through the study (see Figure 1C). Additionally, Model 3b revealed interactions between psychological factors and total time a participant stayed in the study (between-subject time effect). Model 3b revealed a significant interaction between within-subject stress levels and the duration of total time participants remained in the study (OR 1.02, 95% CI 1.01-1.04; *P*>.001). While the main effect of within-subject momentary stress was negatively associated with EMA completion, participants who remained in the study longer exhibited a less pronounced negative impact on survey completion behavior compared to those who dropped out at an earlier stage (see Figure 1D).

## Discussion

### Principal Findings

This study examined factors influencing EMA completion in a 12-month intensive mutiburst longitudinal study among young adults. Our findings reveal a complex interplay of time-varying temporal, contextual, behavioral, and psychological factors and time-invariant individual characteristics that affect EMA completion over time.

Our findings revealed an interesting pattern regarding phone-related factors. While having the phone screen on at the moment of prompt delivery substantially increased the odds of EMA completion, greater phone usage in the prior hour predicted lower completion odds. This seemingly contradictory result may reflect different aspects of device engagement. Having the screen on at prompt delivery represents immediate accessibility and attention to the device, facilitating a quick response. In contrast, higher prior usage may indicate engagement with competing activities on the phone (eg, social media, games, and work tasks) that could reduce willingness to interrupt these activities to complete a survey. Additionally, higher prior usage might indicate a pattern of more fragmented attention or task-switching, making participants less likely to commit to completing the multiquestion EMA. This pattern suggests that EMA designs might benefit from leveraging moments when the device is already in use, while being mindful that excessive prior engagement with the device might create competing demands on user attention.

The observed seasonal effects on completion rates, with higher odds of completion in spring and winter compared to summer, likely reflect behavioral patterns related to indoor or outdoor activity. Research has shown that physical activity levels vary seasonally, which could affect availability for completing assessments [26]. During spring and winter, participants may spend more time indoors, where their phones are more readily accessible and there are fewer competing activities. In contrast, summer months often involve more outdoor activities, travel, and social events where participants may be physically separated from their devices or less willing to interrupt activities to respond to prompts. These seasonal variations could also be attributed to changes in daily routines, such as academic schedules for students or shifts in work patterns [27]. Seasonal changes in mood, particularly seasonal affective disorder, might impact participants' willingness to respond to EMA prompts [28]. Environmental factors like daylight hours and weather conditions have also been found to influence smartphone usage patterns [29], potentially affecting EMA completion rates. Further, the lower completion odds observed outside the home environment, particularly at work or school, highlight the impact of daily routines and competing demands on participants' ability or willingness to respond to prompts. These findings align with previous studies that have identified location and daily activities as important factors in EMA completion [1,7]. Understanding these influences could help researchers design more adaptive EMA protocols that account for predictable fluctuations in participant engagement throughout the year.

Behavioral and psychological states also significantly influenced the odds of EMA completion. Short sleep duration on the previous night and higher levels of physical activity in the preceding 10 minutes were associated with lower completion odds, suggesting that fatigue and engagement in other activities may impede EMA participation. These findings are consistent with previous research indicating that sleep and activity patterns can affect EMA completion [13]. Specifically, studies have shown that poor sleep quality and shorter sleep duration are associated with lower EMA compliance rates [13,30]. This may be due to increased fatigue or cognitive impairment following insufficient sleep, which can reduce participants' motivation or ability to respond to prompts. Regarding physical activity, higher levels of activity, especially during vigorous exercise, have been associated with decreased likelihood of responding to EMA prompts [7,31]. This could be due to the physical unavailability to interact with devices during exercise or the disruption of attention to prompts during activity engagement. Momentary stress was negatively associated with EMA completion rates. The negative association with stress aligns with previous research suggesting that heightened stress can reduce compliance with study protocols [30]. These results underscore the complex relationship between momentary psychological states and EMA engagement, highlighting the need for further research in this area.

Individual characteristics, including sex, race, ethnicity, education, and employment status, were found to be significant predictors of EMA completion. These findings highlight concerns about accessibility and representation when designing and implementing longitudinal multiburst EMA studies, as they may influence participants' ability or willingness to engage with the protocol over time. The observed differences in completion rates across demographic groups align with previous research indicating disparities in EMA compliance [12]. Specifically, studies have found lower compliance rates among racial and ethnic minorities [12,13] and individuals with lower socioeconomic status [32]. Gender differences have also been observed, with some studies reporting lower compliance among males [30], although findings on gender disparities are mixed and may depend on the study context and population.

The decline in completion rates over the course of the study, as indicated by the significant within-subject effect of time, is a common challenge in longitudinal EMA research [1,12]. This decline may reflect participant fatigue, waning motivation, or changes in the perceived value of participation over time [13,33]. The interaction effects observed between time in study and other predictors provide valuable insights into how the influence of various factors on EMA completion evolves throughout a long-term study. For example, the diminishing effect of phone screen status on completion rates over time suggests that participants may become less responsive to this cue as the study progresses [34]. Similarly, the changing relationship between time in study and psychological factor (stress) indicates that the impact of momentary states on compliance may shift as participants become more accustomed to the EMA protocol [30]. The observed interactions between time spent in the study and various predictors, including sex and psychological states, further emphasize the dynamic nature of EMA engagement over extended study periods. These interactions suggest that the factors influencing compliance may shift over time, potentially due to changes in participant motivation, study fatigue, or evolving life circumstances [35].

While within-subject effects of time reveal important patterns in individual trajectories of EMA completion, the between-subject time in study interactions warrant separate consideration. These interactions provide insights into differences between participants who remain in the study for longer periods versus those who drop out earlier. The interaction with stress levels suggests that participants who remained in the study longer were better able to maintain compliance despite experiencing stress. These findings highlight the complex relationship between participant retention, EMA completion, and psychological factors, emphasizing the need to consider both short-term fluctuations and long-term engagement patterns when interpreting EMA data [1,36].

### Limitations

Several limitations should be considered when interpreting these results. First, the study sample was limited to young adults in the United States, potentially limiting generalizability to other age groups or cultural contexts. Second, the use of Android smartphones exclusively may have introduced selection bias and limits the applicability of findings to users of other mobile platforms. Third, the COVID-19 pandemic occurred during the study period, potentially influencing participants' daily routines and psychological states in ways that may not be representative of typical conditions. Additionally, while our models accounted for a wide range of factors, unmeasured variables may still have influenced EMA completion odds. These could include individual differences in personality traits (eg, conscientiousness), technology literacy, or specific life events not captured by our assessments. The use of single-item measures for some psychological constructs (eg, affect and stress) may not fully capture the complexity of these states. Finally, due to the large number of observations per person, this study had statistical power to detect very small effects. While statistically significant, these small effects may not be substantially or clinically meaningful, and their practical implications should be interpreted cautiously. Finally, given the large number of predictors tested in our models, it is important to note that some of the findings may be spurious due to multiple testing. While we have confidence in our overall results, individual predictor effects, especially those with borderline significance, should be interpreted with caution and validated in future studies.

### Implications

These findings have important implications for the design and implementation of future EMA studies. Researchers should consider tailoring prompt schedules to individual participants' routines and contexts to maximize completion rates, as suggested by Nahum-Shani et al [10]. However, it is crucial to recognize that overly tailored designs may yield less representative data if prompts are only delivered during convenient times. This could potentially bias the sample toward certain contexts or psychological states, undermining the ecological validity of the data. Therefore, a balance must be struck between optimizing completion rates and maintaining the representativeness of the collected data. The development of adaptive sampling strategies that account for participants' current state and context could help maintain engagement over extended study periods, but these strategies should be designed to ensure adequate sampling across various contexts and states. The development of adaptive sampling strategies that account for participants' current state and context could help maintain engagement over extended study periods [37]. The observed demographic differences in completion rates suggest that targeted strategies may be necessary to ensure equitable representation in EMA data collection. These strategies may include providing additional support or incentives for groups with lower completion rates or developing culturally sensitive approaches to EMA implementation.

The strong influence of contextual factors such as phone use on EMA completion odds highlights the potential for leveraging smartphone features to enhance EMA engagement. For example, researchers might explore the use of smartwatch-based prompts or other wearable devices to increase the accessibility of EMA surveys [34]. The findings regarding psychological states and completion rates suggest that researchers should carefully consider the timing and frequency of prompts, potentially incorporating measures of participant burden or adjusting protocols based on reported stress levels.

### Conclusions

This study provides valuable insights into the factors influencing EMA completion in a year-long multiburst intensive longitudinal study. The findings highlight the complex and dynamic nature of participant engagement with EMA protocols, emphasizing the need for thoughtful study design and analysis to maximize data quality and representativeness. The observed interactions between time in study and various predictors underscore the importance of considering temporal dynamics in EMA research, particularly for long-term studies. Future research should continue to explore strategies for optimizing EMA methodologies, such as adaptive sampling techniques and the integration of passive sensing data, to enhance our understanding of real-time experiences and behaviors in naturalistic settings. By addressing the challenges identified in this study, researchers can work toward developing more effective and sustainable EMA protocols, ultimately improving the quality and utility of intensive longitudinal data in behavioral science research.

## Appendix

Multimedia Appendix 1 Flowchart describing participant exclusions.

## Abbreviations

DBSCAN: density-based spatial clustering of applications with noise
EMA: ecological momentary assessment
MIMS: monitor-independent movement summary
OR: odds ratio
TIME: Temporal Influences on Movement and Exercise
USC: University of Southern California
