# Supplementary material for: Understanding Longitudinal Ecological Momentary Assessment Completion: Results From 12 Months of Burst Sampling in the TIME Study
Source: JMIR Mhealth Uhealth. 2025 Oct 22;13:e67117. doi: 10.2196/67117 (PMC12590043; doi:10.2196/67117)
Supplement: Multimedia Appendix 1 [file mhealth_v13i1e67117_app1.docx]

**Supplemental Figure 1.** Flowchart describing participant exclusions


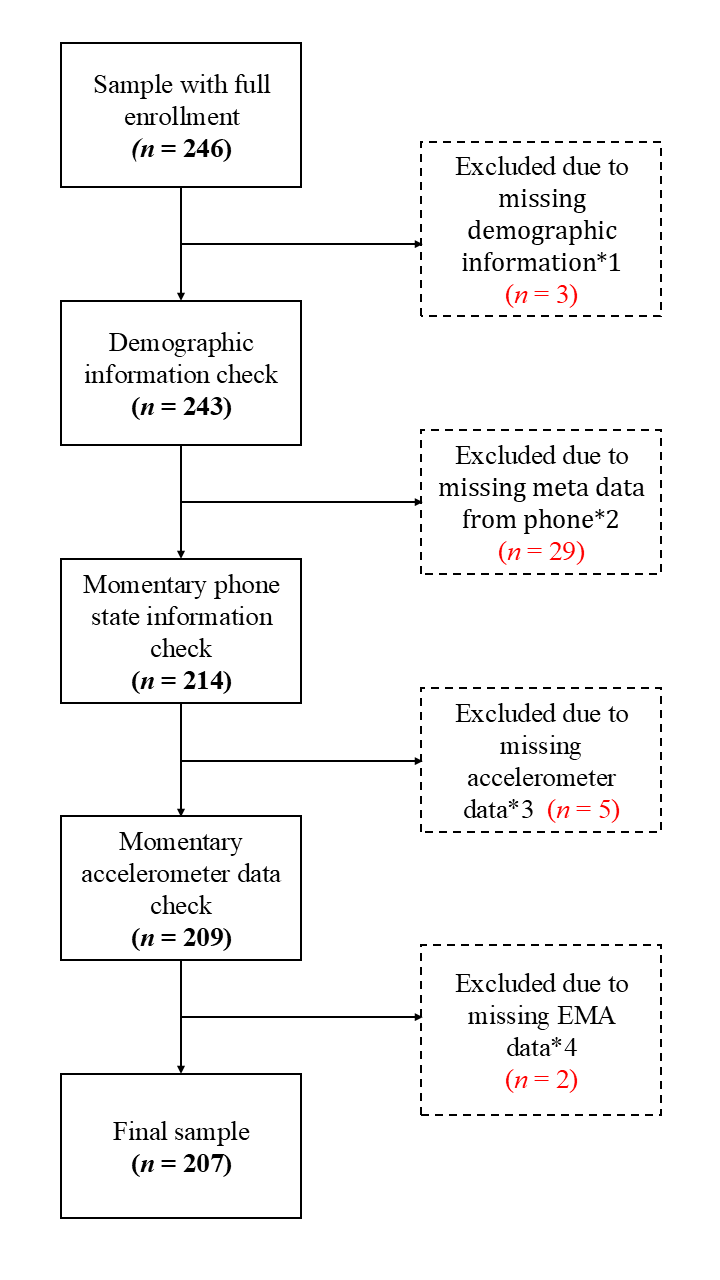


Note: This flowchart outlines the stepwise exclusion of participants due to missing data across multiple data sources. (1) Participants with missing data on key demographic variables—sex at birth and age—were excluded from the analytic sample. (2) Participants with missing data on phone meta data—screen state and phone usage—were excluded from the analytic sample. Screen state refers to status that the participant’s phone screen is on before answering the survey. Phone usage refers to the amount of time phone was “on” estimated based on phone data in an hour before receiving EMA survey. (3) Participants with missing data on accelerometer data—MIMS unit—were excluded from the analytic sample. (4) Participants with missing data on EMA data—responses of momentary positive affect and stress—were excluded from the analytic sample.
